# Cost-effectiveness of fuzuloparib, with or without apatinib, for BRCA mutation HER2-negative metastatic breast cancer in China

**DOI:** 10.3389/fphar.2026.1864488

**Published:** 2026-07-02

**Authors:** Yingzhou Fu, Zhengxiong Li, Qiao Xia, Xiaojuan Chen, Zuojuan Xiang

**Affiliations:** 1 Department of Pharmacy, The Affiliated Cancer Hospital of Xiangya School of Medicine, Central South University, Hunan Cancer Hospital, Changsha, China; 2 School of Medical Informatics and Engineering, Xuzhou Medical University, Xuzhou, China

**Keywords:** apatinib, BRCA mutation, breast cancer, cost-effectiveness, fuzuloparib

## Abstract

**Background:**

The FABULOUS trial showed that fuzuloparib, with or without apatinib, significantly improved progression-free survival (PFS) in patients with human epidermal growth factor receptor 2-negative metastatic breast cancer with germline breast cancer susceptibility genes 1 or 2 mutations compared to standard chemotherapy. The study assessed the cost-effectiveness of three treatment options from the perspective of China’s healthcare system.

**Methods:**

A partitioned survival model was created to evaluate cost-effectiveness. The model incorporated direct medical costs, with drug prices derived from local charges and other costs as well as utility data from published literature. Both costs and utility values were discounted at an annual rate of 4.5%. The incremental cost-effectiveness ratio (ICER) served as the primary outcome. The treatment regimen was determined to be cost-effective if the ICER was below the willingness-to-pay (WTP) threshold of $26,083/quality-adjusted life year (QALY). One-way sensitivity, probabilistic sensitivity and scenario analyses were also conducted.

**Results:**

The ICERs for fuzuloparib versus chemotherapy, fuzuloparib versus fuzuloparib-apatinib, and fuzuloparib-apatinib versus chemotherapy were calculated as $141,912.74/QALY, $285,196.26/QALY, and $90,074.03/QALY, respectively. Accordingly, neither fuzuloparib plus apatinib nor fuzuloparib monotherapy was found to be cost-effective compared with standard chemotherapy at the WTP threshold of $26,083/QALY. Moreover, fuzuloparib monotherapy was not cost-effective compared with fuzuloparib plus apatinib. Sensitivity and scenario analyses affirmed the robustness of the model. One-way sensitivity analysis showed that utility for PFS, utility for progressive disease, and costs associated with subsequent treatment exerted the most substantial influence on the outcomes.

**Conclusion:**

Although fuzuloparib, with or without apatinib, has shown substantial clinical benefits, it has not proven to be cost-effective when compared to standard chemotherapy. Further global multicenter or population-specific clinical trials involving breast cancer patients are necessary to enhance the understanding of the application scope and cost-effectiveness of fuzuloparib.

## 1 Introduction

Breast cancer ranks first in terms of incidence, mortality, and disability-adjusted life years among female cancers globally, imposing a substantial social and economic burden (Bray et al., 2024; Li T. et al., 2025). In 2022, China reported 357,200 new cases of breast cancer and 75,000 related deaths (Han et al., 2024), with the majority of diagnoses occurring in women aged 45–49 (Qian et al., 2023). The financial burden of breast cancer treatment in China amounted to 3.8 billion dollars in 2018, representing 6.4% of the total expenditure on cancer treatment (Zhou et al., 2024). Furthermore, the presence of relapse or metastasis and an advanced clinical stage at diagnosis were correlated with significantly elevated treatment costs (Wu et al., 2024). Among breast cancer patients, 5%–10% are attributed to hereditary factors, with germline breast cancer susceptibility genes 1 or 2 (BRCA1/2) mutations being the most prevalent genetic predisposition (Pohl-Rescigno et al., 2020; Kuchenbaecker et al., 2017). The proteins encoded by BRCA1 and BRCA2 are integral to the repair of DNA double-strand breaks via the homologous recombination repair pathway (Walsh, 2015). Conversely, the enzyme poly (adenosine diphosphate–ribose) polymerase (PARP) is involved in the repair of DNA single-strand breaks. Cells exhibiting functional deficiencies in BRCA1 or BRCA2 are particularly susceptible to PARP inhibitors, rendering it a pivotal therapeutic strategy for breast cancer associated with BRCA1/2 mutations (Petropoulos et al., 2024; Wang et al., 2026). Several PARP inhibitors, such as olaparib, talazoparib, and veliparib, have been shown to significantly enhance progression-free survival (PFS) in advanced breast cancer with BRCA1/2 mutations (Robson et al., 2017; Litton et al., 2018; Diéras et al., 2020). However, they failed to demonstrate an improvement in overall survival (OS). In 2024, China granted approval for the use of fuzuloparib, with or without apatinib, for the treatment of germline BRCA-mutated human epidermal growth factor receptor 2 (HER2)-negative metastatic breast cancer. This represents the third global approval of a PARP inhibitor, following olaparib and talazoparib, and the first approval of a PARP inhibitor in China for this specific indication. The approval was predicated on the findings from the FABULOUS trial, which was conducted across 40 centers in China (Li H. et al., 2025). This is the first three-arm phase 3 study to direct compare the PARP inhibitor monotherapy with a combination therapy involving anti-angiogenic agents for advanced breast cancer. It reported a median PFS of 11.0 months for the fuzuloparib-apatinib group, 6.7 months for the fuzuloparib group, and 3.0 months for the chemotherapy group. Notably, the median OS was observed to be 29.2 months, 31.5 months, and 21.5 months, respectively. Although the OS data are not yet mature, both the fuzuloparib-apatinib and fuzuloparib monotherapy groups exhibited favorable benefit trends. According to the 2026 Chinese Society of Clinical Oncology (CSCO) guideline, for patients with germline BRCA mutations and programmed death ligand 1 (PD-L1)-negative advanced triple-negative breast cancer (TNBC) who have not previously responded to taxane-based treatments, fuzuloparib plus apatinib has been recommended as a first-line treatment, while fuzuloparib monotherapy has been advised as a second-line treatment.

It is worth noting that fuzuloparib, with or without apatinib, has been incorporated into the reimbursement framework for metastatic breast cancer treatment in China since 2026. This inclusion in the National Medical Insurance Catalog (NMIC) is expected to enhance its clinical application and address the treatment gap for HER2-negative metastatic breast cancer with germline BRCA mutations. Beyond clinical efficacy and safety, economic evaluations are crucial for health-related decision-making and rational clinical practice. Currently, there is a lack of studies on the cost-effectiveness of fuzuloparib monotherapy, fuzuloparib plus apatinib, and standard chemotherapy. This study aims to assess the cost-effectiveness of these three treatment regimens from the perspective of China’s healthcare system.

## 2 Methods

The research adhered to the Consolidated Health Economic Evaluation Reporting Standards (CHEERS) reporting guideline (Husereau et al., 2022), as detailed in Supplementary Table 1.

### 2.1 Population and interventions

The patient characteristics in this study were delineated with reference to the FABULOUS trial and included the following criteria: female patients aged between 18 and 75 years with pathologically confirmed HER2-negative breast cancer; confirmed or suspected deleterious BRCA1/2 mutations; prior treatment with anthracyclines or taxanes; no more than two lines of treatment in the recurrent or metastatic stage. Patients with hormone receptor-positive status should have received at least one line of endocrine therapy. Additionally, participants were required to have an Eastern Cooperative Oncology Group (ECOG) performance status score of 0–1 and the presence of evaluable lesions as defined by the Response Evaluation Criteria in Solid Tumors (RECIST) version 1.1.

Participants were randomly allocated in a 1:1:1 ratio to receive either fuzuloparib (100 mg taken orally twice a day) plus apatinib (500 mg taken orally once a day), fuzuloparib monotherapy (150 mg taken orally twice a day), or standard chemotherapy. The chemotherapy regimen consisted of either capecitabine (1000–1250 mg/m^2^ taken orally twice a day from days 1–14 in each 21-day cycle) or vinorelbine (25–30 mg/m^2^ given intravenously on days 1 and 8 of each 21-day cycle). Patients in the chemotherapy cohort were permitted to transition to fuzuloparib monotherapy upon disease progression. The FABULOUS trial included patients with TNBC and hormone receptor-positive breast cancer, who had received up to two prior treatments for recurrent or metastatic disease. Consequently, patients received different treatments based on their clinical conditions after progression. To closely match the clinical trial scenario, we determined the proportions of subsequent treatments, including chemotherapy, endocrine therapy, immunotherapy, and targeted therapy, utilizing data from published trials. These regimens were frequently selected in clinical practice for managing HER2-negative metastatic breast cancer. In order to ensure consistency with real-world clinical practice, the specific dosing regimens were aligned with the CSCO guidelines. Comprehensive details and dosing regimens were shown in Supplementary Table 2. The body surface area for dosing purposes was based on the 2020 China National Nutrition and Chronic Disease Status Report (CNNCDSP), which indicated that adult females have an average height of 1.58 m, a weight of 59 kg, and a body surface area of 1.62 m^2^.

### 2.2 Model structure

**FIGURE 1:**
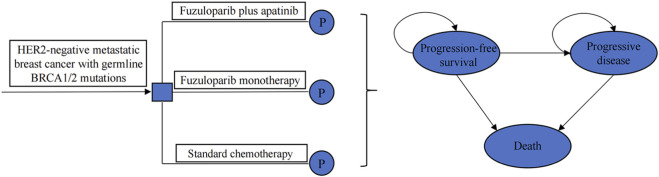
Model structure. P, partitioned survival model.

A partitioned survival model was developed using TreeAge Pro 2022 to assess the cost-effectiveness for patients with HER2-negative metastatic breast cancer carrying germline BRCA mutations. Total cost, quality-adjusted life years (QALYs), and incremental cost-effectiveness ratio (ICER) were the primary outcomes. The model incorporated three mutually exclusive health states: PFS, progressive disease (PD), and death. Patients were assumed to enter the model in the PFS state. Over time, they might either remain in PFS or transition to PD or death, with disease progression being irreversible, as shown in Figure 1. This modeling approach avoids additional assumptions required in state-transition models by directly estimating health state occupancy using an area-under-the-curve method derived from the OS and PFS curves. The analysis was conducted over a 20-year time horizon, during which the mortality rate in all groups surpassed 99%. The model cycle was aligned with the 21-day dosing schedule of the clinical trial. In accordance with the threshold recommendations of the 2025 China Guidelines for Pharmacoeconomic Evaluations (CGPE), and with reference to the willingness-to-pay (WTP) survey findings in the Chinese population reported by Xu et al. (2024), this study adopted 1.94 times China’s 2024 per capita gross domestic product (GDP) as the WTP threshold, equivalent to $26,083/QALY. An ICER value below this WTP threshold signified that the intervention was cost-effective; otherwise, it was not cost-effective.

The proportion of patients within each health status in the model was directly derived from the area under the OS and PFS curves. Consequently, we reconstructed and extrapolated the survival curves utilizing the algorithm proposed by Guyot et al. (2012). Data points were extracted from the original curve using the WebPlotDigitizer software, facilitating the reconstruction of individual patient data (IPD) within the R software. The uncertainty associated with IPD reconstruction can be exacerbated during long-term extrapolation, resulting in increased variability in predictive outcomes. Consequently, the IPDfromKM package was employed to verify the accuracy of curve reconstruction by comparing the reconstructed IPD with the original survival data. The IPD was fitted and extrapolated using a range of models, including standard survival models (exponential, Weibull, gamma, log-normal, log-logistic, Gompertz, generalized gamma) and flexible models (fractional polynomial [FP], restricted cubic spline [RCS], and Royston-Parmar [RP] spline) (Shao et al., 2023a). The FP models encompassed both first-order and second-order optimal models, while the RP models assessed the optimal models for odds, normal, and hazard scales. The Akaike Information Criterion (AIC) and log-likelihood (LnL) were computed to evaluate the goodness of fit. Concurrently, visual inspection and clinical rationality were used as additional criteria to determine the optimal survival model. Specifically, the optimal model should adequately capture the tail characteristics of the survival curve, and the PFS probability must consistently remain less than or equal to the OS probability. This approach is particularly helpful when the AIC or LnL values of competing models are closely aligned, facilitating the selection of the most plausibility survival model. The fitting results for each model were presented in Supplementary Figures 1–6; Supplementary Table 3. The optimal models and their key parameters were detailed in Table 1.

**TABLE 1:** Optimal and sub-optimal distribution key parameters for survival curves.

Survival curve	Optimal model	Key parameters	Sub-optimal model	Key parameters
Fuzuloparib-apatinib group-OS	RP-normal-2	k = 2, gamma0 = −0.5237, gamma1 = 2.0054, gamma2 = 2.3325, gamma3 = −2.5332	RP-normal-1	k = 0, gamma0 = −1.0236, gamma1 = 1.1696
Fuzuloparib-apatinib group-PFS	Log-logistic	shape = 0.6313, scale = −0.1406	RP-odds-1	k = 0, gamma0 = 0.2643, gamma1 = 1.8801
Fuzuloparib group-OS	Log-normal	meanlog = 0.8625, sdlog = −0.1484	RP-hazard-2	k = 3, gamma0 = 1.1357, gamma1 = 6.9553, gamma2 = 9.5376, gamma3 = −18.7491, gamma4 = 13.1024
Fuzulopari group-PFS	RP-normal-1	k = 5, gamma0 = 5.4545, gamma1 = 3.7606, gamma2 = 11.4963, gamma3 = −27.7555, gamma4 = 29.2794, gamma5 = −7.4089, gamma6 = 3.0088	RP-normal-2	k = 0, gamma0 = 0.6833, gamma1 = 1.0580
Chemotherapy group-OS	RP-odds-2	k = 2, gamma0 = 0.2685, gamma1 = 2.6120, gamma2 = 1.0848, gamma3 = −1.4346	FP1-2	intercept = −0.2615, I (time^(-0.5)^) = -0.5326
Chemotherapy group-PFS	RP-hazard-1	k = 3, gamma0 = 14.9486, gamma1 = 7.6314, gamma2 = 16.4746, gamma3 = −6.9287, gamma4 = 1.8923	Log-normal	meanlog = −1.2453, sdlog = −0.1505

FP, fractional polynomials; LnL, log-likelihood; OS, overall survival; PFS, progression-free survival; RCS, restricted cubic splines; RP, Royston-Parmar.

### 2.3 Cost and utility

The model was conducted from the perspective of China’s healthcare system, focusing solely on direct medical costs. These costs encompassed imaging assessments, laboratory tests, best supportive care, terminal care, medication, intravenous drug administration, and the management of grade≥3 adverse events (AEs), as detailed in Table 2. Based on clinical trial protocols, imaging assessments were conducted bi-cyclically during the first ten cycles, followed by tri-cyclical assessments thereafter. It was presumed that each patient received best supportive care after disease progression and terminal care before death. Drug pricing data were obtained from the Hunan Provincial Medical Security Bureau database to represent the drug prices in China’s public hospitals. For drugs produced by multiple manufacturers, we calculated the unit price of each manufacturer’s product using an appropriate standard dosage and then took the average value. As grade 1–2 AEs are well-managed, only costs related to grade ≥3 AEs were considered in the model. Using the Consumer Price Index, all costs were brought to the 2024 level and converted to US dollars at an exchange rate of 1 USD to 7.1217 RMB.

**TABLE 2:** Key model parameters.

Parameter	Base-case value (range)	Distribution	References
Cost ($)			
Imaging evaluation per time	85.01 (13.91 ∼ 309.12)	Gamma	Shu et al. (2025)
Laboratory test per cycle	77.29 (61.83 ∼ 92.75)	Gamma	Pan et al. (2024)
Best supported care per cycle	189.28 (151.42 ∼ 227.14)	Gamma	Pan et al. (2024)
Terminal care	2,461.86 (1,969.49 ∼ 2,954.23)	Gamma	Shu et al. (2025)
Intravenous infusion per unit	7.10 (5.68 ∼ 8.51)	Gamma	Shu et al. (2025)
Fuzuloparib 50 mg	7.25 (5.80 ∼ 8.70)	Gamma	Local charge
Apatinib 500 mg	24.80 (19.84 ∼ 29.76)	Gamma	Local charge
Capecitabine 1000 mg	1.42 (0.50 ∼ 5.69)	Gamma	Local charge
Vinorelbine 10 mg	20.71 (7.72 ∼ 37.57)	Gamma	Local charge
Decreased white blood cells	446.05 (356.84 ∼ 535.26)	Gamma	Shu et al. (2025)
Neutrophil count decreased	446.05 (356.84 ∼ 535.26)	Gamma	Shu et al. (2025)
Anaemia	976.43 (781.14 ∼ 1,171.72)	Gamma	Shu et al. (2025)
Hypertension	14.64 (11.71 ∼ 17.57)	Gamma	Dai et al. (2024)
Platelet count decreased	500.00 (400.00 ∼ 600.00)	Gamma	Shu et al. (2025)
Hypertriglyceridaemia	4.63 (3.70 ∼ 5.56)	Gamma	Ding et al. (2025)
Lymphocyte count decreased	446.05 (356.84 ∼ 535.26)	Gamma	Shu et al. (2025)
Cost of subsequent treatment in fuzuloparib-apatinib group per cycle	1,872.46 (1,497.97 ∼ 2,246.95)	Gamma	Estimated
Cost of subsequent treatment in fuzuloparib group per cycle	2,471.32 (1,977.06 ∼ 2,965.58)	Gamma	Estimated
Cost of subsequent treatment in chemotherapy group per cycle	1,002.22 (801.78 ∼ 1,202.66)	Gamma	Estimated
Utility			
PFS	0.87 (0.70 ∼ 1.00)	Beta	Zhu et al. (2023) Xu et al. (2016)
PD	0.71 (0.57 ∼ 0.85)	Beta	Zhu et al. (2023) Xu et al. (2016)
Disutility			
Decreased white blood cells	0.09 (0.07 ∼ 0.11)	Beta	Shu et al. (2025)
Neutrophil count decreased	0.10 (0.08 ∼ 0.12)	Beta	Shu et al. (2025)
Anaemia	0.19 (0.15 ∼ 0.23)	Beta	Shu et al. (2025)
Hypertension	0.04 (0.03 ∼ 0.05)	Beta	Dai et al. (2024)
Platelet count decreased	0.20 (0.16 ∼ 0.24)	Beta	Shu et al. (2025)
Hypertriglyceridaemia	0.15 (0.12 ∼ 0.18)	Beta	Ding et al. (2025)
Lymphocyte count decreased	0.09 (0.07 ∼ 0.11)	Beta	Shu et al. (2025)
Risk of grade≥3 AEs in fuzuloparib-apatinib group			
Decreased white blood cells	0.07 (0.06 ∼ 0.08)	Beta	Li et al. (2025a)
Neutrophil count decreased	0.13 (0.10 ∼ 0.16)	Beta	Li et al. (2025a)
Anaemia	0.11 (0.09 ∼ 0.13)	Beta	Li et al. (2025a)
Hypertension	0.13 (0.10 ∼ 0.16)	Beta	Li et al. (2025a)
Platelet count decreased	0.10 (0.08 ∼ 0.12)	Beta	Li et al. (2025a)
Hypertriglyceridaemia	0.09 (0.07 ∼ 0.11)	Beta	Li et al. (2025a)
Lymphocyte count decreased	0.03 (0.02 ∼ 0.04)	Beta	Li et al. (2025a)
Risk of grade≥3 AEs in fuzuloparib group			
Decreased white blood cells	0.19 (0.15 ∼ 0.23)	Beta	Li et al. (2025a)
Neutrophil count decreased	0.20 (0.16 ∼ 0.24)	Beta	Li et al. (2025a)
Anaemia	0.37 (0.30 ∼ 0.44)	Beta	Li et al. (2025a)
Hypertension	0 (NA)	Beta	Li et al. (2025a)
Platelet count decreased	0.12 (0.10 ∼ 0.14)	Beta	Li et al. (2025a)
Hypertriglyceridaemia	0.01 (0.01 ∼ 0.01)	Beta	Li et al. (2025a)
Lymphocyte count decreased	0.10 (0.08 ∼ 0.12)	Beta	Li et al. (2025a)
Risk of grade≥3 AEs in chemotherapy group			
Decreased white blood cells	0.18 (0.14 ∼ 0.22)	Beta	Li et al. (2025a)
Neutrophil count decreased	0.24 (0.19 ∼ 0.29)	Beta	Li et al. (2025a)
Anaemia	0.08 (0.06 ∼ 0.10)	Beta	Li et al. (2025a)
Hypertension	0 (NA)	Beta	Li et al. (2025a)
Platelet count decreased	0 (NA)	Beta	Li et al. (2025a)
Hypertriglyceridaemia	0.02 (0.02 ∼ 0.02)	Beta	Li et al. (2025a)
Lymphocyte count decreased	0.03 (0.02 ∼ 0.04)	Beta	Li et al. (2025a)
Body surface area, m^2^	1.62 (1.30 ∼ 1.94)	Normal	CNNCDSP
Discount rate (%)	4.50 (0 ∼ 5.00)	Fixed	CGPE

AE, adverse event; CGPE, china guidelines for pharmacoeconomic evaluations; CNNCDSP, china national nutrition and chronic disease status report; PD, progressive disease; PFS, progression-free survival.

While the FABULOUS trial reported the mean scores of the EORTC QLQ-C30 for each group, it did not provide sufficient data to calculate utility values. As a result, this study utilized health utility values, calculated using the EQ-5D-5L scale for Chinese breast cancer patients, from similar literature (Zhu et al., 2023; Xu et al., 2016). To more accurately capture the impact of each group’s safety profile on the outcomes, the disutility associated with grade ≥3 AEs was also incorporated, as shown in Table 2. Both costs and utility values were discounted at an annual rate of 4.5% according to the CGPE.

### 2.4 Sensitivity analysis

A one-way sensitivity analysis was employed to assess the impact of variations in a single variable on the model outcomes. The range for each variable was sourced from existing literature or set at ±20% of the baseline value (Shu et al., 2025). In cases where a drug had multiple manufacturers, the price variation range was determined by the maximum and minimum values. This methodology ensured comprehensive coverage of the effects of low-cost drugs, particularly in the context of China’s centralized procurement policy, as well as the influence of higher-priced originator drugs on the results. Since the subsequent treatment regimens assumed after disease progression were hardly to fully align with actual data, we increased their fluctuation range to ±30%. The outcomes of the one-way sensitivity analysis were illustrated using a tornado diagram. Additionally, a probabilistic sensitivity analysis was conducted by assigning an appropriate distribution to each variable and performing 1,000 Monte Carlo simulations to evaluate the combined effects of all variables changing simultaneously on the results. The findings from this analysis were presented using a cost-effectiveness acceptability curve and an ICER scatter plot.

### 2.5 Scenario analysis

In scenario 1, we aligned each protocol’s treatment duration with the median duration from the FABULOUS trial: 5 cycles for the chemotherapy group, 8 cycles for the fuzuloparib group, and 13 cycles of fuzuloparib plus 10 cycles of apatinib for the fuzuloparib-apatinib group. This scenario offered a significant reference point for simulating the variability in real-world treatment discontinuation times, since some patients may be unable to maintain medication adherence until disease progression. Since the utility values were derived from the literature, we replaced them in scenario 2 by setting the utility of PFS to 0.81 and the utility of PFS to 0.68. These values were also calculated based on the EQ-5D-5L scale for patients with advanced breast cancer, but not for the Chinese population (Cameron et al., 2023). In scenario 3 and scenario 4, the model time horizon was set to 5 years and 10 years, respectively, to evaluate the impact of the simulated duration on the model. To evaluate the impact of uncertainty stemming from the fitting and extrapolation of survival curves to the model, a sub-optimal distribution was applied to each curve in scenario 5, as detailed in Table 1.

## 3 Results

### 3.1 Base-case analysis

In the base-case analysis, the costs associated with the fuzuloparib group, the fuzuloparib-apatinib group, and the chemotherapy group were $119,383.95, $79,429.85, and $44,551.25, respectively. The corresponding QALYs for these groups were 2.08, 1.94, and 1.55, respectively. The ICERs for fuzuloparib versus chemotherapy, fuzuloparib versus fuzuloparib-apatinib, and fuzuloparib-apatinib versus chemotherapy were calculated as $141,912.74/QALY, $285,196.26/QALY, and $90,074.03/QALY, respectively. As shown in Table 3. As a result, neither fuzuloparib plus apatinib nor fuzuloparib monotherapy was found to be cost-effective compared with standard chemotherapy when the WTP threshold was set at $26,083/QALY. Moreover, fuzuloparib monotherapy was not cost-effective compared with fuzuloparib plus apatinib.

**TABLE 3:** The results of base-case and scenario analyses.

Group	Total cost ($)	QALYs	ICER ($/QALY, pairwise comparison)	
Base-case analysis				
Chemotherapy group	44,551.25	1.55	-	-
Fuzuloparib-apatinib group	79,429.85	1.94	90,074.03	-
Fuzuloparib group	119,383.95	2.08	141,912.74	285,196.26
Scenario 1				
Chemotherapy group	44,044.38	1.55	-	-
Fuzuloparib-apatinib group	65,550.85	1.94	55,540.48	-
Fuzuloparib group	111,629.97	2.08	128,169.31	328,917.23
Scenario 2				
Chemotherapy group	44,551.25	1.48	-	-
Fuzuloparib-apatinib group	79,429.85	1.83	99,505.07	-
Fuzuloparib group	119,383.95	1.97	152,186.83	282,970.73
Scenario 3				
Chemotherapy group	39,268.68	1.39	-	-
Fuzuloparib-apatinib group	73,869.14	1.80	83,781.14	-
Fuzuloparib group	98,018.95	1.73	170,699.83	−350,947.09
Scenario 4				
Chemotherapy group	43,532.83	1.52	-	-
Fuzuloparib-apatinib group	79,204.38	1.93	86,956.91	-
Fuzuloparib group	115,838.04	2.02	144,877.46	412,274.88
Scenario 5				
Chemotherapy group	42,109.99	1.46	-	-
Fuzuloparib-apatinib group	94,157.38	2.23	67,566.96	-
Fuzuloparib group	220,469.31	3.53	86,163.28	97,184.91

ICER, incremental cost-effectiveness ratio; QALY, quality-adjusted life year.

### 3.2 Sensitivity analysis

**FIGURE 2:**
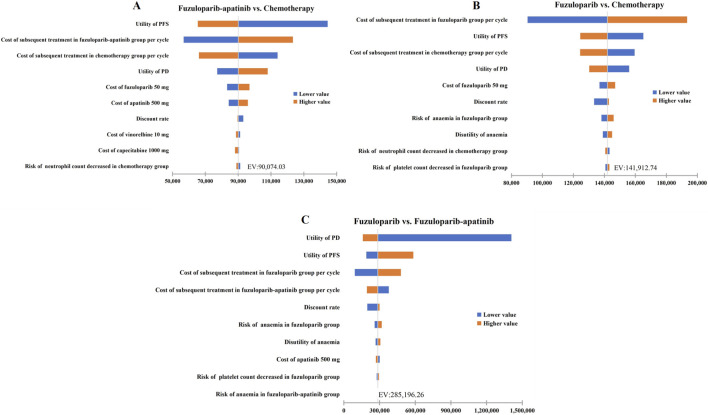
Tornado diagram of one-way sensitivity analysis results. **(A)** Fuzuloparib-apatinib vs. Chemotherapy. **(B)** Fuzuloparib vs. Chemotherapy. **(C)** Fuzuloparib vs. Fuzuloparib-apatinib. PD, progressive disease; PFS, progression-free survival.

**FIGURE 3:**
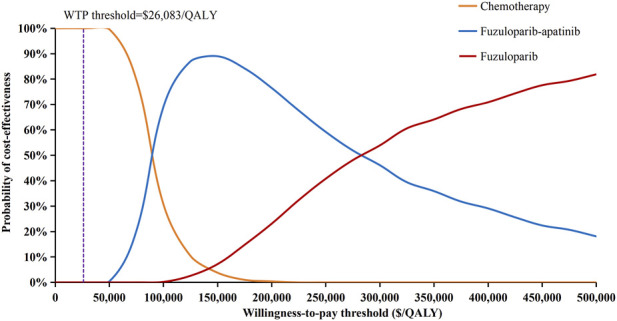
Cost-effectiveness acceptability curve of probabilistic sensitivity analysis results. QALYs, quality-adjusted life-years; WTP, willingness-to-pay.

**FIGURE 4:**
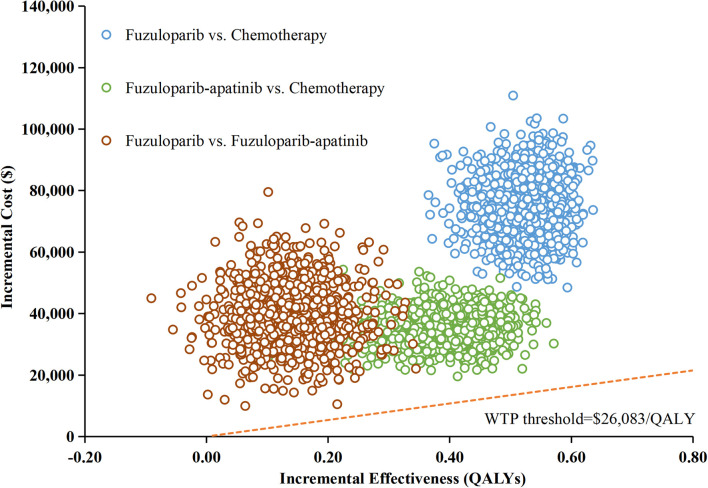
ICER scatter plot of probabilistic sensitivity analysis results. QALYs, quality-adjusted life-years; WTP, willingness-to-pay.

Figure 2 illustrated the top ten variables influencing outcomes in the tornado diagram. Among these variables, the utility of PFS, the utility of PD, and the cost of subsequent treatment exert the most substantial impact on cost-effectiveness. Nonetheless, no variable within the predefined fluctuation range reduced the ICER value below the WTP threshold. The cost-effectiveness acceptability curve depicted in Figure 3 indicated that as the WTP threshold increases, the likelihood of chemotherapy being the most cost-effective option gradually diminished. Figure 4 displayed ICER scatter plots. Simulation points located above the WTP threshold line indicated that the intervention was not cost-effective. The findings demonstrated that, under the current WTP threshold, pairwise comparisons confirmed a 100% probability of maintaining the economic ranking order of the three treatment options in the base-case analysis.

### 3.3 Scenario analysis

The results of the scenario analysis were displayed in Table 3. Adjustments to the number of treatment cycles based on median treatment duration, modifications to utility values, selection of sub-optimal models for each survival curve, or alterations to the model’s time horizon did not affect the cost-effectiveness results, thereby demonstrating the robustness of the model.

## 4 Discussion

PARP inhibitors provide a crucial precision treatment option for breast cancer patients with BRCA1/2 mutations (Wang et al., 2025). Although fuzuloparib monotherapy or its combination with apatinib has demonstrated significant clinical advantages over standard chemotherapy, our study indicated that neither treatment achieved cost-effectiveness at the WTP threshold of $26,083/QALY. Nonetheless, due to their significant clinical impact, fuzuloparib and apatinib have been incorporated into the 2026 NMIC at their current pricing. This inclusion suggests that, from the healthcare system’s perspective, addressing the unmet need for later-line treatment options in BRCA1/2 mutation HER2-negative metastatic breast cancer, the annual budgetary impact of providing these medications to the relatively small population with BRCA mutations may be manageable. The reduction in out-of-pocket expenses facilitates better access to targeted therapies, enabling a broader patient population to benefit from the clinical advantages of innovative treatments. This is expected to promote equity in healthcare resources and mitigate disparities in diagnosis and treatment across diverse regions and demographic groups. Concurrently, the economic evaluation results emphasize the necessity of comprehensively assessing both the potential clinical benefits and the patients’ financial capacity when formulating individualized appropriate treatment plans. Furthermore, the study by Nie et al. demonstrated that for patients in China with germline BRCA1/2 mutations and platinum-sensitive recurrent ovarian carcinoma, fuzuloparib was cost-effective when compared to niraparib, but less so when compared to olaparib (Pan et al., 2024). Research by Pan et al. suggested that talazoparib was a cost-effective alternative to standard treatment for germline BRCA1/2 mutated HER2-negative advanced breast cancer in China (Nie et al., 2023). However, it is important to note that this indication for talazoparib has not yet received approval.

Fuzuloparib plus apatinib demonstrated superior cost-effectiveness compared to fuzuloparib monotherapy. The improved PFS might be one of the contributors to this outcome. Mechanistically, anti-angiogenic agents possess the ability to downregulate homologous recombination repair genes, thereby exhibiting synergistic effects when combined with PARP inhibitors (Ray-Coquard et al., 2019; Bindra et al., 2005; Lim et al., 2014). However, the median OS in the fuzuloparib-apatinib group was slightly lower than that observed in the fuzuloparib monotherapy group. In scenario analysis, when the time horizon was reduced to 5 years, the cost-effectiveness of the fuzuloparib-apatinib combination was accentuated, and it became the dominant strategy, with an ICER of $-350,947.09/QALY. In contrast, extending the time horizon to 10 or 20 years revealed a gradual emergence of the potential benefits of fuzuloparib monotherapy in terms of OS, leading to an increase in QALYs and surpassing those of the fuzuloparib-apatinib group, thereby rendering the ICER positive. However, this did not alter the overall economic profile of the two treatment strategies. Besides the effectiveness range, the cost also contributes to ICER. Although apatinib increased the risk of hypertension (Qin et al., 2021), the combination therapy cost less in AE management. This is because the fuzuloparib plus apatinib therapy led to fewer serious AEs, possibly due to lower dosages. The rate of serious AEs was 12.9%, compared to 17.9% in the fuzuloparib group and 13.6% in the chemotherapy group (Li H. et al., 2025). Furthermore, it also demonstrated reduced rates of hematologic toxicity, the management of which was typically associated with substantial costs. Consequently, the safety benefits of the fuzuloparib plus apatinib regimen translated into notable cost advantages in the cost-effectiveness analysis. These variations in AE rates also underscore the importance of considering safety as equally crucial as clinical benefits and cost-effectiveness when choosing personalized treatment plans.

One-way sensitivity analysis revealed that the cost of subsequent treatment significantly influenced the economic outcomes. Nevertheless, even when the fluctuation range was extended to ±30%, the model results remained stable. The lower cost of subsequent treatment in the chemotherapy group was due to the fact that patients were permitted to receive fuzuloparib monotherapy after disease progression, as per the FABULOUS trial. This substantially reduced the proportion of patients choosing other costly targeted therapies. Furthermore, this finding suggested that further clarification of subsequent treatment regimens and their sequence of administration for such patients could enhance the reliability of cost-effectiveness outcomes in the future. Additionally, the utility values for PFS and PD had a remarkable impact on the model. However, changes in the utility values in the scenario analysis also did not alter the economic ranking of the three treatment options.

This is the first study to assess the cost-effectiveness of fuzuloparib, with or without apatinib, for BRCA mutation HER2-negative metastatic breast cancer. The primary strength of this study lied in its utilization of the partitioned survival model, a methodology particularly well-suited for the development of tumor-related models compared to the Markov model (Pan et al., 2024; Xu et al., 2025). Given the availability of survival curves, this methodology facilitates direct modeling based on reconstructed survival data, thereby ensuring a high degree of alignment with actual clinical data. Moreover, the FABULOUS trial was conducted in China, which reduced the impact of regional and ethnic heterogeneity on the model. Furthermore, the study employed both standard parametric models and flexible models to fit survival curves. This strategy effectively addressed the limitations associated with capturing inflection points in survival curves when using standard parametric models, as well as the risk of overfitting linked to flexible models, both of which could potentially compromise the accuracy of extrapolations (Shao et al., 2023a; Shao et al., 2023b). To further assess the impact of curve extrapolation uncertainty on the model, a scenario analysis was conducted using sub-optimal distributions, and the results demonstrate the robustness of the model’s outcomes.

This study also had several limitations. Firstly, while the study established the economic ranking of three treatments in China, it remains uncertain whether this ranking is applicable to other breast cancer sub-populations or other regions. In particular, the cost parameters and survival data of the model may need adjustment when the background changes. For instance, in patients with BRCA mutations or homologous recombination proficient ovarian cancer, the combination of fuzuloparib and apatinib did not demonstrate additional PFS benefits compared to fuzuloparib alone; however, the combination regimen proved superior to fuzuloparib monotherapy in patients with homologous recombination deficiency (Wu et al., 2026). Moreover, the FABULOUS trial only reported limited subgroup survival data, which was insufficient for a rigorous subgroup cost-effectiveness evaluation. Well designed clinical trials would be highly beneficial for further exploring the application scope and cost-effectiveness of fuzuloparib. Secondly, despite optimizing the fitting and extrapolation method to address structural uncertainty and conducting corresponding scenario analyses, it is crucial to validate the model with real-world data in the future, especially as the OS data of the fuzuloparib-apatinib and fuzuloparib group were immature. Thirdly, the assumed subsequent treatment regimens may not fully align with actual data. Therefore, further validation using real-world evidence would enhance the model’s reliability. Fourthly, the model incorporated only the costs and disutility associated with grade 3–4 AEs, which could potentially introduce bias. Nonetheless, the one-way sensitivity analysis indicated that variables related to AEs had a minimal impact on the model’s outcomes. Lastly, this study did not consider the influence of regional economic disparities in China on the findings. For example, the drug prices, medical service costs, and the WTP threshold based on *per capita* GDP utilized in this study may not be representative or applicable to specific regions.

## 5 Conclusion

While fuzuloparib, with or without apatinib, has shown significant clinical benefits for patients with HER2-negative metastatic breast cancer harboring germline BRCA mutations, it has not demonstrated cost-effectiveness in comparison to chemotherapy in China. Moreover, fuzuloparib plus apatinib is more cost-effective than fuzuloparib alone. Conducting rigorously designed global multicenter or population-specific breast cancer patient clinical trials in the future will enhance the understanding of the application scope and cost-effectiveness of fuzuloparib.

## Data Availability

The original contributions presented in the study are included in the article/Supplementary Material, further inquiries can be directed to the corresponding author.
