# Supplementary material for: Cost-effectiveness of fuzuloparib, with or without apatinib, for BRCA mutation HER2-negative metastatic breast cancer in China
Source: Front Pharmacol. 2026 Jul 2;17:1864488. doi: 10.3389/fphar.2026.1864488 (PMC13373583; doi:10.3389/fphar.2026.1864488)
Supplement: Supplementary file 1 [file DataSheet1.PDF]

Supplementary Material

1 Supplementary Tables

Supplementary TABLE 1. CHEERS Checklist (2022)

Topic	No.	Item	Reported?
**Title**			
	1	Identify the study as an economic evaluation and specify the interventions being compared.	Yes
**Abstract**			
	2	Provide a structured summary that highlights context, key methods, results, and alternative analyses.	Yes
**Introduction**			
**Background and objectives**	3	Give the context for the study, the study question, and its practical relevance for decision making in policy or practice.	Yes
**Methods**			
**Health economic analysis plan**	4	Indicate whether a health economic analysis plan was developed and where available.	Not applicable
**Study population**	5	Describe characteristics of the study population (such as age range, demographics, socioeconomic, or clinical characteristics).	Yes
**Setting and location**	6	Provide relevant contextual information that may influence findings.	Yes
**Comparators**	7	Describe the interventions or strategies being compared and why chosen.	Yes
**Perspective**	8	State the perspective(s) adopted by the study and why chosen.	Yes
**Time horizon**	9	State the time horizon for the study and why appropriate.	Yes
**Discount rate**	10	Report the discount rate(s) and reason chosen.	Yes

Topic	No.	Item	Reported?
**Selection of outcomes**	11	Describe what outcomes were used as the measure(s) of benefit(s) and harm(s).	Yes
**Measurement of outcomes**	12	Describe how outcomes used to capture benefit(s) and harm(s) were measured.	Yes
**Valuation of outcomes**	13	Describe the population and methods used to measure and value outcomes.	Yes
**Measurement and valuation of resources and costs**	14	Describe how costs were valued.	Yes
**Currency, price date, and conversion**	15	Report the dates of the estimated resource quantities and unit costs, plus the currency and year of conversion.	Yes
**Rationale and description of model**	16	If modelling is used, describe in detail and why used. Report if the model is publicly available and where it can be accessed.	Yes
**Analytics and assumptions**	17	Describe any methods for analysing or statistically transforming data, any extrapolation methods, and approaches for validating any model used.	Yes
**Characterising heterogeneity**	18	Describe any methods used for estimating how the results of the study vary for subgroups.	Not applicable
**Characterising distributional effects**	19	Describe how impacts are distributed across different individuals or adjustments made to reflect priority populations.	Not applicable
**Characterising uncertainty**	20	Describe methods to characterise any sources of uncertainty in the analysis.	Yes
**Approach to engagement with patients and others affected by the study**	21	Describe any approaches to engage patients or service recipients, the general public, communities, or stakeholders (such as clinicians or payers) in the design of the study.	Not applicable
**Results**			
**Study parameters**	22	Report all analytic inputs (such as values, ranges, references) including uncertainty or distributional assumptions.	Yes
**Summary of main results**	23	Report the mean values for the main categories of costs and outcomes of interest and summarise them in the most appropriate overall measure.	Yes

Topic	No.	Item	Reported?
**Effect of uncertainty**	24	Describe how uncertainty about analytic judgments, inputs, or projections affect findings. Report the effect of choice of discount rate and time horizon, if applicable.	Yes
**Effect of engagement with patients and others affected by the study**	25	Report on any difference patient/service recipient, general public, community, or stakeholder involvement made to the approach or findings of the study	Not applicable
**Discussion**			
**Study findings, limitations, generalisability, and current knowledge**	26	Report key findings, limitations, ethical or equity considerations not captured, and how these could affect patients, policy, or practice.	Yes
**Other relevant information**			
**Source of funding**	27	Describe how the study was funded and any role of the funder in the identification, design, conduct, and reporting of the analysis	Yes
**Conflicts of interest**	28	Report authors conflicts of interest according to journal or International Committee of Medical Journal Editors requirements.	Yes

Supplementary TABLE 2. Usage and dosage of the subsequent treatment.

Treatment strategy	Dosing schemes					
	Fuzuloparib-apatinib group	Rate	Fuzuloparib group	Rate	Chemotherapy group	Rate
Chemotherapy	Either capecitabine 1000–1250 mg/m² orally twice daily on days 1–14 of each 21-day cycle; Or nab-paclitaxel 100–150 mg/m² intravenously on days 1, 8, 15 of each 21-day cycle	83.33%	Either capecitabine 1000–1250 mg/m² orally twice daily on days 1–14 of each 21-day cycle; Or nab-paclitaxel 100–150 mg/m² intravenously on days 1, 8, 15 of each 21-day cycle	83.78%	Either capecitabine 1000–1250 mg/m² orally twice daily on days 1–14 of each 21-day cycle; Or nab-paclitaxel 100–150 mg/m² intravenously on days 1, 8, 15 of each 21-day cycle	40.00%
Endocrine therapy	Anastrozole 1 mg orally once daily	33.33%	Anastrozole 1 mg orally once daily	40.54%	Anastrozole 1 mg orally once daily	22.50%
Immunotherapy	Toripalimab 240 mg intravenously on day 1 each 21-day cycle	13.33%	Toripalimab 240 mg intravenously on day 1 each 21-day cycle	21.62%	Toripalimab 240 mg intravenously on day 1 each 21-day cycle	12.50%
Targeted therapy	Either abemaciclib 150 mg orally twice daily, or sacituzumab govitecan 10 mg/kg on days 1 and 8 of each 21-day cycle	40.00%	Either abemaciclib 150 mg orally twice daily, or sacituzumab govitecan 10 mg/kg on days 1 and 8 of each 21-day cycle	54.05%	Either abemaciclib 150 mg orally twice daily, or sacituzumab govitecan 10 mg/kg on days 1 and 8 of each 21-day cycle, or fuzuloparib 150 mg orally twice daily	95.00%

Drug price: $1.42/capecitabine 1000mg, $0.52/nab-paclitaxel 1 mg, $0.56/anastrozole, $1.18/toripalimab 1mg, $9.8/abemaciclib 150mg, $6.55/sacituzumab govitecan 1mg, $7.25/fuzuloparib 50mg.

Supplementary TABLE 3. Summary of statistical goodness-of-fit of the standard and flexible parametric models.

Model	Fuzuloparib-apatinib group-OS		Fuzuloparib-apatinib group-PFS		Fuzuloparib group-OS		Fuzulopari group-PFS		Chemotherapy group-OS		Chemotherapy group-PFS	
	LnL	AIC	LnL	AIC	LnL	AIC	LnL	AIC	LnL	AIC	LnL	AIC
Exponential	-49.215	100.430	-59.706	121.412	-46.705	95.410	-54.462	110.925	-45.944	93.888	-42.872	87.744
Weibull	-44.822	93.645	-57.535	119.071	-43.487	90.974	-53.695	111.390	-44.121	92.241	-42.247	88.493
Gamma	-44.453	92.906	-57.029	118.058	-42.809	89.619	-53.143	110.286	-43.994	91.988	-41.260	86.520
Log-normal	-43.888	91.776	-56.844	117.688	-41.618	87.235	-51.274	106.549	-43.888	91.775	-36.087	76.174
Gompertz	-46.314	96.627	-59.034	122.069	-45.530	95.059	-54.453	112.906	-44.678	93.355	-42.760	89.521
Log-logistic	-44.555	93.110	-56.448	116.896	-42.478	88.957	-51.568	107.136	-44.219	92.438	-37.678	79.356
Generalized gamma	-43.745	93.491	-56.696	119.392	-40.904	87.807	-51.761	109.522	-43.829	93.658	NA	NA
FP1-1	-47.806	99.612	-60.235	124.469	-46.754	97.508	-55.081	114.163	-44.673	93.345	-42.952	89.904
FP1-2	-44.316	92.631	-57.193	118.385	-42.603	89.206	-53.215	110.430	-43.609	91.218	-41.390	86.779
FP2-1	-46.054	98.107	-58.544	123.088	-42.726	91.452	-54.948	115.896	-44.530	95.060	-42.053	90.106
FP2-2	-45.840	97.681	-57.974	121.948	-41.991	89.983	-54.512	115.025	-44.562	95.123	-41.603	89.207
RCS1	-46.081	98.163	-57.708	121.416	-41.990	89.981	-54.152	114.305	-44.561	95.122	-41.638	89.275
RCS2	-45.150	98.300	-57.205	122.410	-41.404	90.808	-51.748	111.495	-43.590	95.180	-39.989	87.978
RP-hazard-1	-42.405	92.810	-56.706	119.411	-40.835	87.670	-49.469	112.937	-44.121	92.241	-32.522	75.044
RP-hazard-2	-44.822	93.645	-57.535	119.071	-39.404	88.808	-51.869	109.737	-43.811	93.623	-34.558	81.116
RP-odds-1	-42.243	92.486	-56.448	116.896	-40.844	87.689	-49.441	112.882	-44.219	92.438	-35.077	78.153
RP-odds-2	-44.555	93.110	-56.498	118.996	-39.515	89.031	-51.568	107.136	-43.337	94.674	-35.642	83.284
RP-normal-1	-43.888	91.776	-56.844	117.688	-41.618	87.235	-49.283	112.565	-43.888	91.775	-35.578	79.156
RP-normal-2	-42.014	92.029	-56.529	119.058	-40.861	87.722	-51.274	106.549	-43.849	93.698	-47.940	107.880

AIC, Akaike Information Criterion; FP, Fractional Polynomials; LnL, log-likelihood; OS, overall survival; PFS, progression-free surviva; RCS, Restricted Cubic Splines; RP, Royston-Parmar.

2 Supplementary Figures

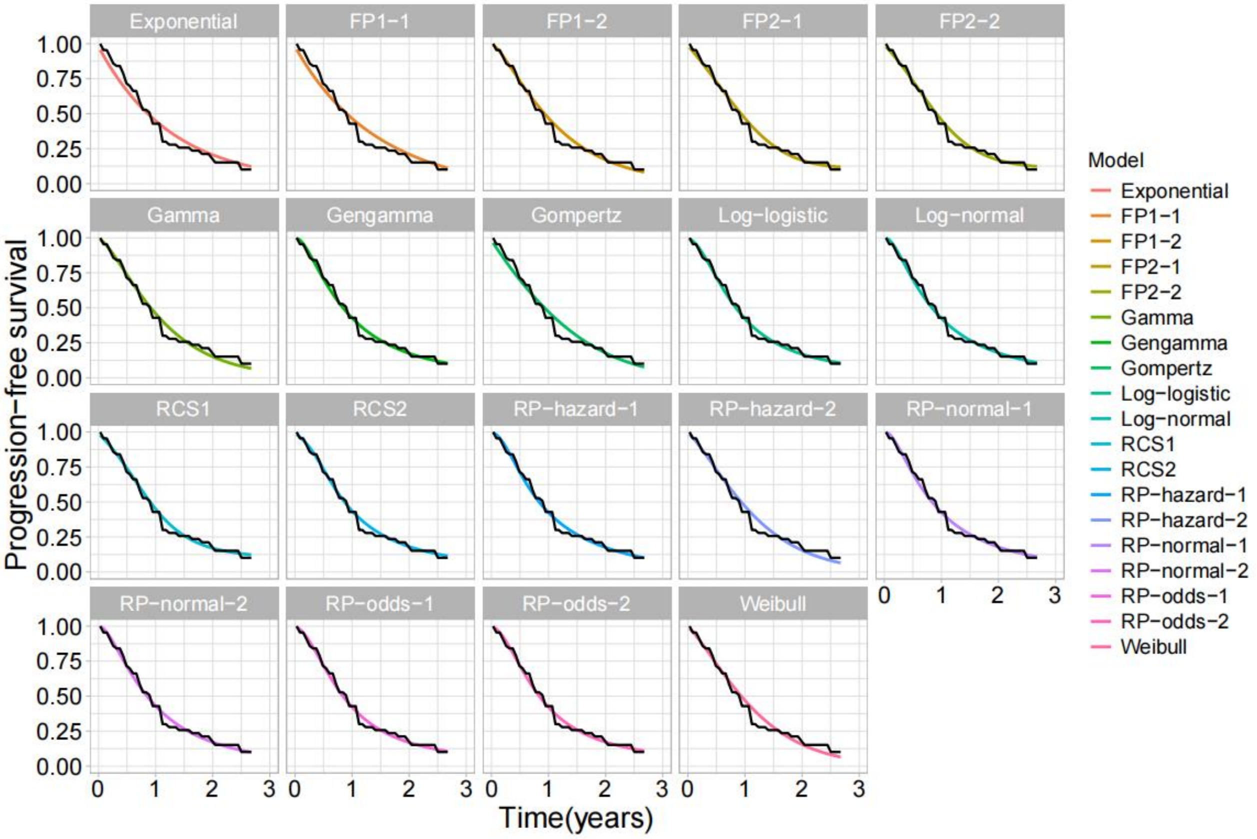

Supplementary FIGURE 1. Fitting of the progression-free survival curve for the fuzuloparib-apatinib group. FP, Fractional Polynomials; RCS, Restricted Cubic Splines; RP, Royston-Parmar.

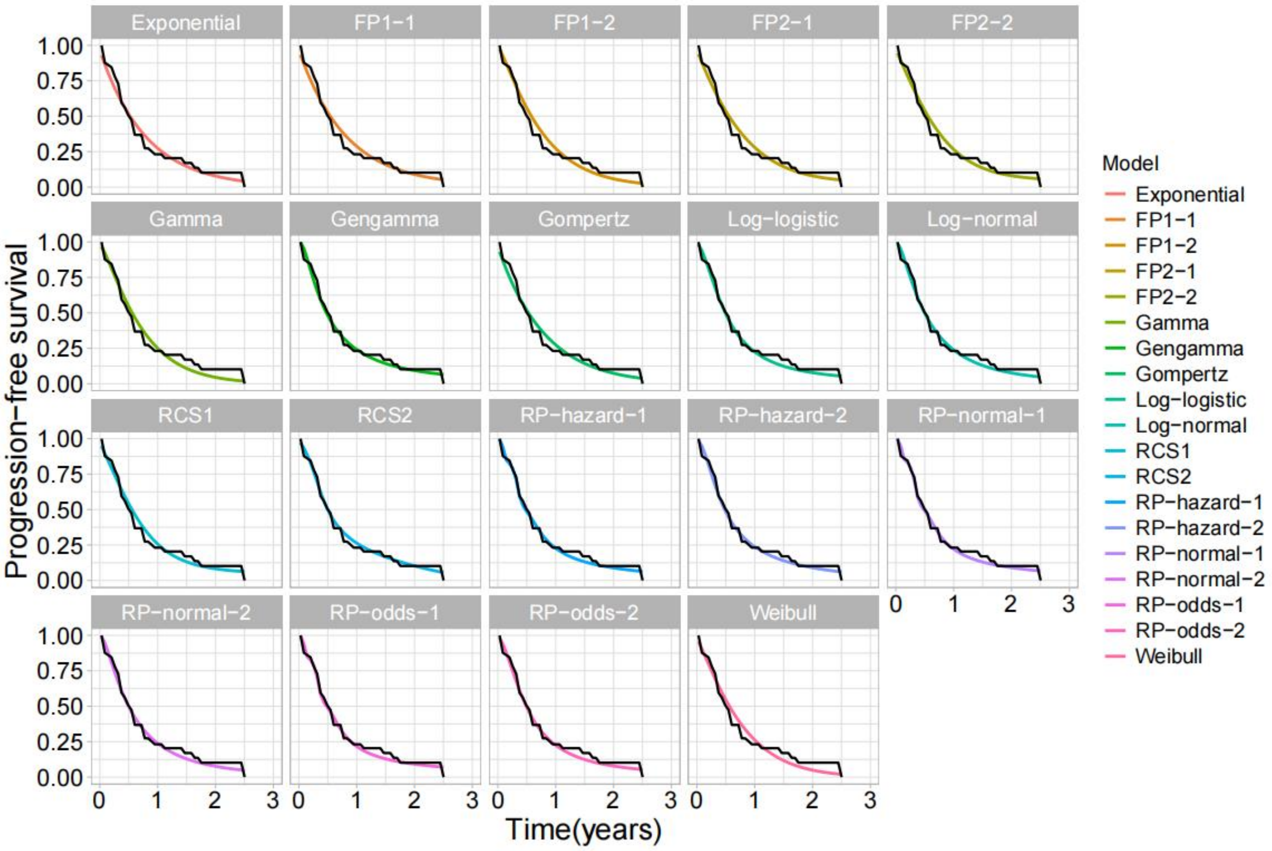

Supplementary FIGURE 2. Fitting of the progression-free survival curve for the fuzuloparib group. FP, Fractional Polynomials; RCS, Restricted Cubic Splines; RP, Royston-Parmar.

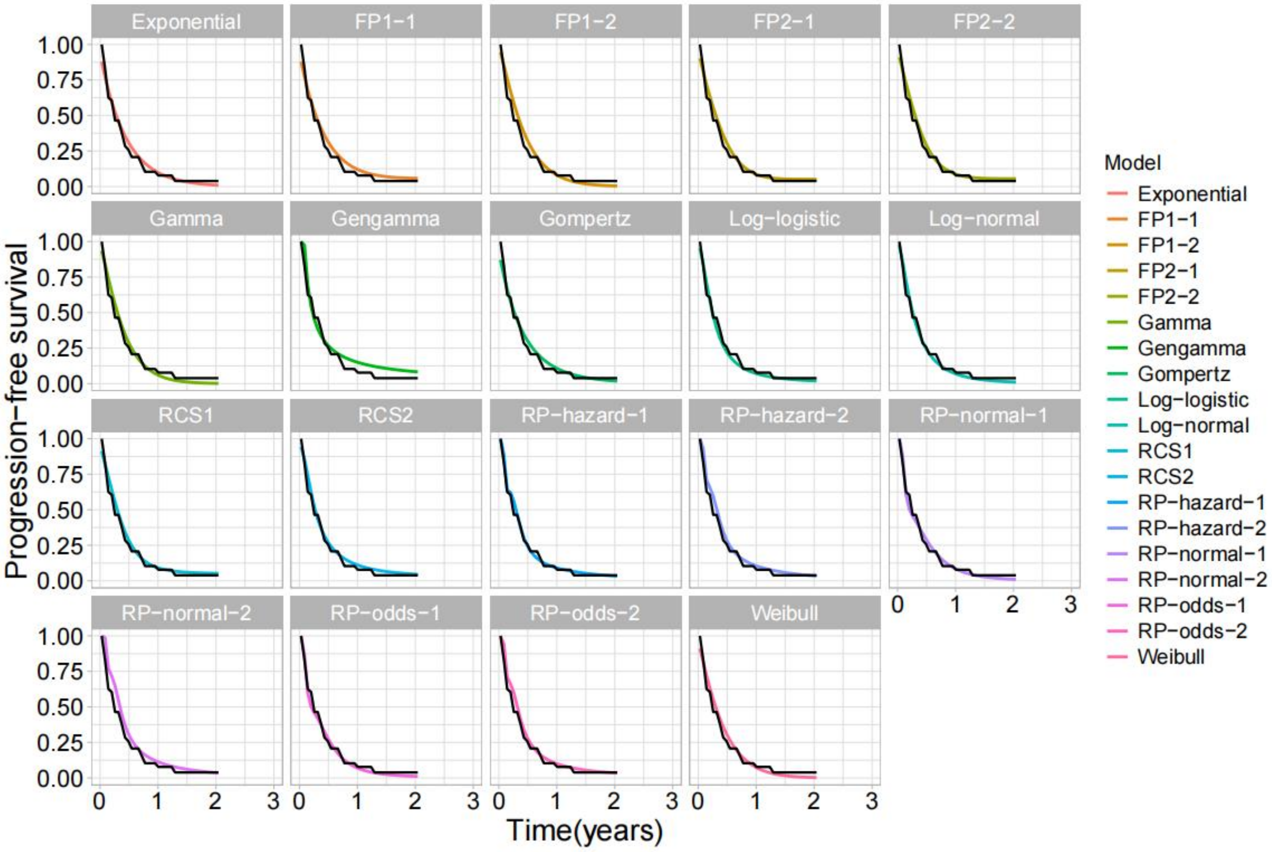

Supplementary FIGURE 3. Fitting of the progression-free survival curve for the chemotherapy group. FP, Fractional Polynomials; RCS, Restricted Cubic Splines; RP, Royston-Parmar.

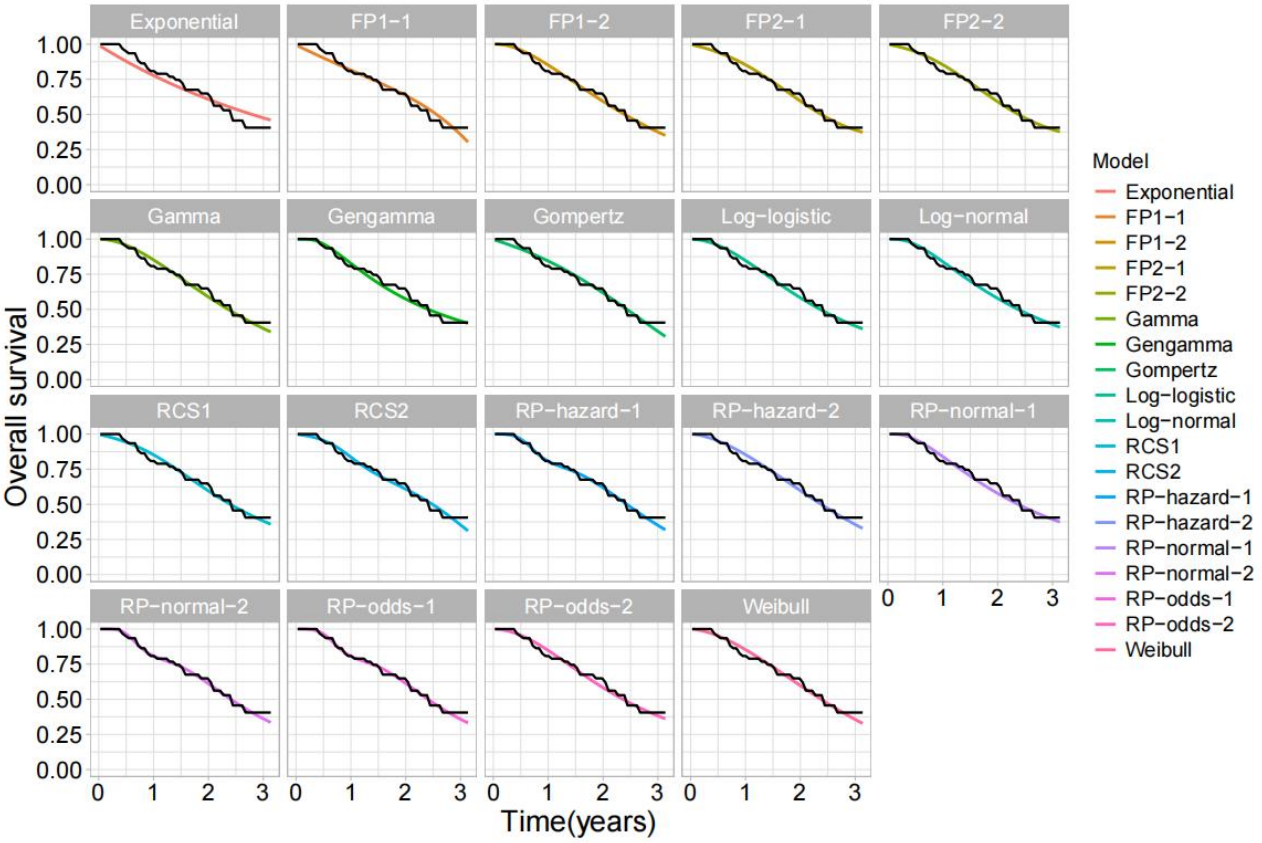

Supplementary FIGURE 4. Fitting of the overall survival curve for the fuzuloparib-apatinib group. FP, Fractional Polynomials; RCS, Restricted Cubic Splines; RP, Royston-Parmar.

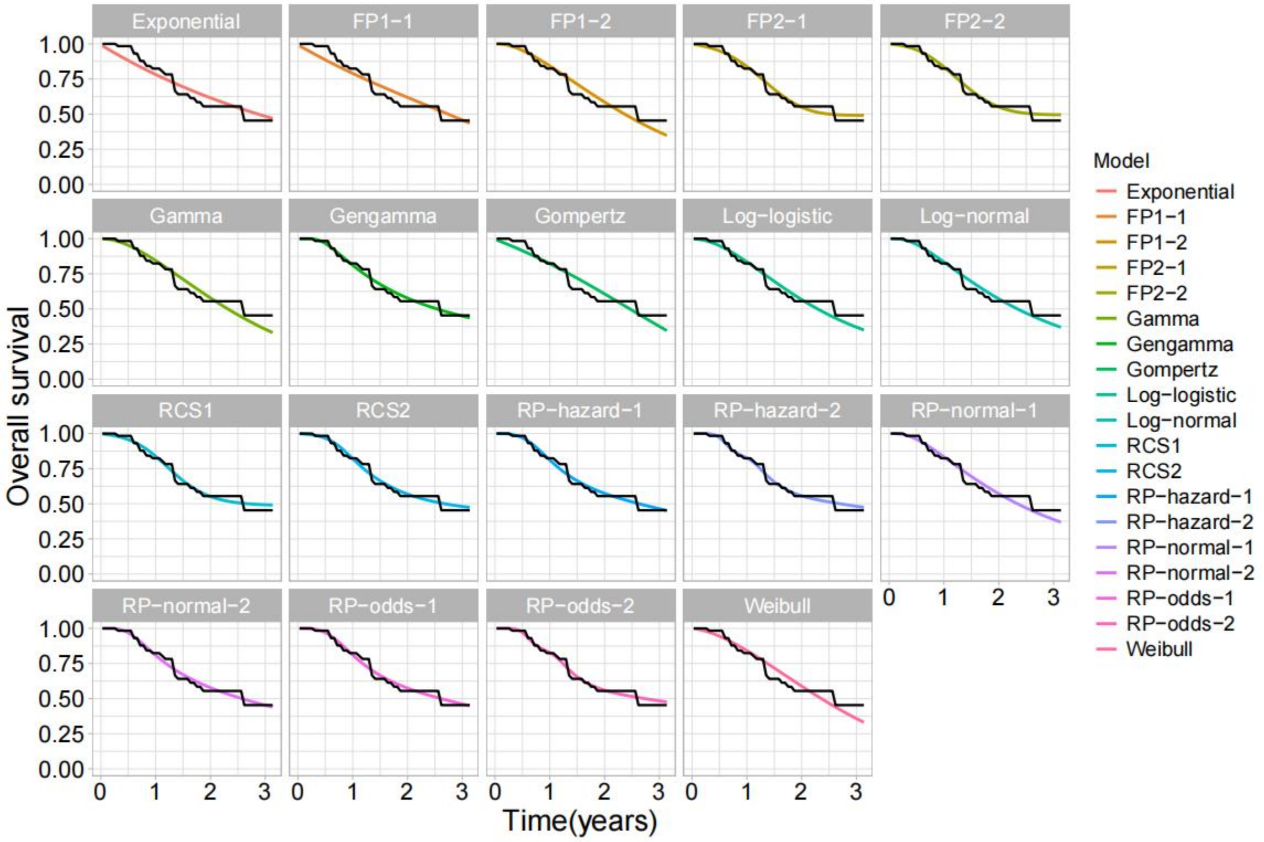

Supplementary FIGURE 5. Fitting of the overall survival curve for the fuzuloparib group. FP, Fractional Polynomials; RCS, Restricted Cubic Splines; RP, Royston-Parmar.

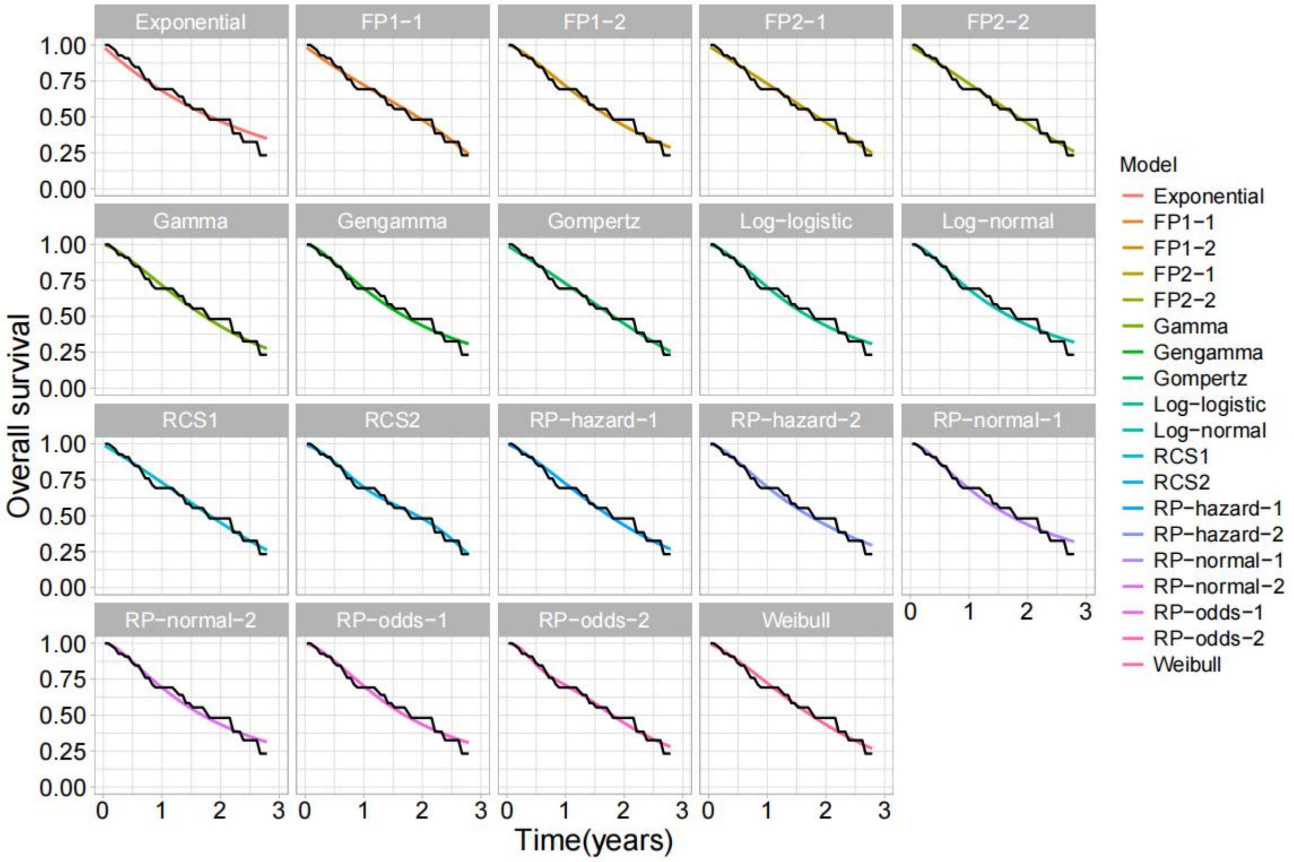

Supplementary FIGURE 6. Fitting of the overall survival curve for the chemotherapy group. FP, Fractional Polynomials; RCS, Restricted Cubic Splines; RP, Royston-Parmar.
